# Scheduling rules for patients with diabetes mellitus that facilitate split-dosing improve the quality of bowel preparation for colonoscopy

**DOI:** 10.1371/journal.pone.0182225

**Published:** 2017-07-31

**Authors:** Robert J. Hilsden, Ronald Bridges, Catherine Dube, Steven J. Heitman, Alaa Rostom

**Affiliations:** 1 Department of Medicine, Cumming School of Medicine, University of Calgary, Calgary, Alberta, Canada; 2 Department of Community Health Sciences, Cumming School of Medicine, University of Calgary, Calgary, Alberta, Canada; 3 University of Ottawa, Ottawa, Ontario, Canada; University Hospital Llandough, UNITED KINGDOM

## Abstract

**Background & aims:**

An adequate bowel preparation for colonoscopy is best achieved by giving the cleansing regimen as a split-dose with the second dose given 4–6 hours before the procedure. This can be difficult to administer to diabetics who are preferentially scheduled for early morning procedures. We examined the impact on bowel preparation quality of scheduling diabetics for mid-morning (9:30 am or later) procedures rather than early morning procedures (7:30–9:00 AM) to facilitate a split-dose preparation.

**Methods:**

Historical cohort study of 34,415 patients (1,805 diabetics) age 18–74 years without significant comorbidities who underwent an outpatient colorectal cancer screening-related colonoscopy either before (2013) or after (2014) a unit wide change in scheduling practices for diabetics. The primary outcome was the rate of inadequate bowel preparation. Secondary outcomes include the rate of procedures complete to the cecum, procedure duration and detection rates of polyps, any colorectal cancer screening-relevant lesion (adenoma, sessile serrated adenoma, large proximal hyperplastic polyp) and advanced adenomas.

**Results:**

From 2013 to 2014, the proportion of diabetics with an inadequate bowel preparation decreased from 7.7% to 3.2% (95% confidence interval for the difference 2.2%–6.8%, P<0.00005). There was no significant change in the proportion of non-diabetics with inadequate preparation (2% in both years). There was no change in secondary outcomes in diabetics from 2013 to 2014.

**Conclusions:**

Preferentially scheduling diabetic patients later in the morning that more conveniently allowed for a split dose bowel preparation resulted in decreased rates of inadequate bowel preparation without disadvantaging other patients.

## Introduction

Patients with diabetes mellitus are one of several groups who are at increased risk for poor bowel preparation when undergoing colonoscopy.[1–5] High quality bowel preparation is critically important for the detection of adenomatous and proximal serrated polyps and the application of guideline-based recommendations for colorectal cancer screening intervals.[6–8]

In 2014, the US Multi-Society Task Force on Colorectal Cancer provided evidenced-based recommendations to optimize colonoscopy preparation quality and patient safety.[9] Foremost among the recommendations made by the Task Force was the use of a split-dose bowel cleansing regimen, with the second dose of the preparation beginning 4–6 hours before the time of the colonoscopy. However, implementing this recommendation for early morning procedures is challenging as it requires the patient to start the second dose of the preparation around 2:30 AM for a 7:30 AM procedure. Therefore, endoscopy units often default to administering all of the preparation the day before the procedure for early morning procedures.

Patients with diabetes mellitus are routinely recommended to undergo procedures early in the morning to minimize the duration of time that they must fast.[10] At our unit, a quality improvement audit of bowel preparation quality identified that diabetics, who were routinely given early morning appointments, were less likely to have an adequate preparation than other patients. In response to this finding, the unit changed the scheduling of diabetic patients from early morning procedures (before 9:30 am) to mid-morning (9:30 am or later) to allow them to routinely complete a split dose preparation.

The purpose of this study was to examine the impact of scheduling patients with diabetes mellitus for mid-morning procedures with a split-dose preparation rather than for an early morning procedure with a day-before preparation. The primary outcome was the rate of inadequate bowel preparation. We also examined secondary outcomes including procedure duration, depth of insertion and detection rates for CRC screening-relevant lesions.

## Methods

### Study design and patients

This study received IRB approval by the Conjoint Health Research Ethics Board of the University of Calgary (REB17-0116). The study was conducted at the Forzani & MacPhail Colon Cancer Screening Centre in Calgary, AB, Canada. The Centre is a publicly-funded endoscopy unit that provides only screening-related colonoscopies. Colonoscopies performed for other indications, such as the investigation of signs or symptoms of gastrointestinal disease, are performed at hospital endoscopy units. All patients must be free of medical conditions that would place them at higher risk for colonoscopy-related adverse events (ASA Class I/II). This means that any diabetic patient must be free of advanced end-organ complications, such as renal failure or gastroparesis. All patients undergo a consultation appointment with a trained nurse, and those who do not meet the Centre’s eligibility criteria (for example, those with signs or symptoms of gastrointestinal disease) are redirected elsewhere. Colonoscopies at the Centre are performed by gastroenterologists and colorectal surgeons who also perform endoscopies at hospital endoscopy units. Patients are allocated to endoscopy lists from a common queue.

The Centre runs morning lists (starting at 07:30 AM) and afternoon lists (starting at 12:30 PM) of eight colonoscopies scheduled in 30 minute time slots. In November 2013, the scheduling practice of the Centre was changed to preferentially schedule patients with diabetes mellitus at 9:30 AM or later.

In this historical cohort study, we obtained data on 34,533 patients who underwent a colonoscopy at the Centre from January to October 2013 (before scheduling change) and from January to December 2014 (after scheduling change). To be included in the study, a patient had to have undergone a screening-related colonoscopy between the ages of 18 and 76 years. Indications for procedures included average risk for colorectal cancer, personal or family history of colorectal cancer or polyps and positive guaiac fecal occult blood or fecal immunochemical test. The fecal immunochemical test replaced the guaiac fecal occult blood test in Alberta in November 2013. For patients undergoing more than one procedure, only the first procedure was included. Patients were also excluded if information on the quality of the bowel preparation was missing.

The standard bowel preparation used by the Centre during the time of the study was a split-dose four liter polyethylene glycol (PEG) preparation without any adjuncts. Patients scheduled before 9:30 AM received both doses the day prior to the procedure. Patients scheduled 9:30 AM or later received the second 2 liter dose starting 5 hours prior to the scheduled time of their appointment. Patients with a history of allergy or intolerance to PEG-based preparations were offered an alternative, usually a combination of Pico-Salax® (Ferring Phamaceuticals, North York, Canada) and bisacodyl, also administered in a day prior or split dose manner depending on appointment time. In addition, all patients consumed a low fiber diet starting four days before the procedure and stopping after breakfast the day before the procedure. All patients were encouraged to drink clear fluids until two hours before the colonoscopy.

All patients completed an in-person medical assessment and education session prior to the date of their colonoscopy. At that appointment, patients participated in a large group education presentation that included information on preparation of the colon, including the importance of a good preparation for adenoma detection and tips for completing the preparation. After the session, each patient had an individual meeting with an endoscopy nurse who reinforced the importance of the bowel preparation and addressed any concerns or uncertainties of the patient regarding the preparation. Each patient also received detailed written bowel preparation instructions in English and, if appropriate, in one of six languages most common in Calgary. The patients were also given a phone number that they could call if they had any subsequent questions regarding the preparation.

Bowel preparation was rated by the endoscopist using a modification of the “Adequate/Inadequate” scale recommended by the Quality Assurance Task Force of the National Colorectal Cancer Roundtable.[11] This scale is based on the endoscopist’s opinion of whether the examination was adequate to detect lesions larger than 5 mm. With the Centre’s modification, the endoscopist selected one of three ratings for the quality of the bowel preparation rated after any washing and suctioning of residual bowel contests was performed:

1. Adequate–clean: The bowel preparation resulted in a clean colon that required minimal irrigation and/or suctioning of residual bowel contents during the colonoscopy.
2. Adequate–stool: After the bowel preparation, there was residual liquid and/or stool, but this was removed with irrigation and suctioning during the colonoscopy.
3. Inadequate: There was residual liquid and/or stool that could not be adequately cleared with irrigation and suctioning during the colonoscopy.

The Centre’s practices dictated that patients with an inadequate rating would be scheduled for a repeat colonoscopy or, in some cases, for an alternative screening exam (CT colonography, fecal immunochemical test). Those with an adequate rating would receive guideline-based surveillance recommendations for the timing of their next colonoscopy.

### Data sources and variables

We obtained data on colonoscopies from the Centre’s endoscopy reporting program endoPRO™ (Pentax Medical). Data elements included age, gender, presence of diabetes mellitus, procedure date, indication, depth of endoscope insertion, bowel preparation quality, duration of procedure and whether a polypectomy was performed. Pathology data was obtained from the Centre’s Pathology Database, which includes a structured summary of the pathology report. The summary is completed by trained nurses who reconcile each polyp reported at colonoscopy with the pathology report. The nurses also select an appropriate surveillance interval for the patient based on the individual’s underlying colorectal cancer risk (eg average risk) and the colonoscopy results using an algorithm based on the US Multi-Society Task Force on Colorectal Cancer guidelines.[12]

### Statistical analysis

Statistical analysis was performed using Stata 14 (StataCorp LLP, College Station TX). The primary analysis focused on the difference and associated 95% exact confidence interval (CI) for the difference between the 2013 and 2014 rates of inadequate bowel preparation among diabetics. The impact on year of procedure on the rates of inadequate bowel preparation were further examined in diabetics by two separate logistic regression models that included patient age and gender as independent predictor variables.

Secondary analyses examined differences between diabetic patients in 2013 and 2014 in terms of rates of incomplete procedures (cecum not reached), duration of procedure and rates of detection of any polyp, any screen-relevant lesion and any advanced adenoma. A screen-relevant lesion was defined as any adenomatous polyp, sessile serrated adenoma, traditional serrated adenoma, large (> 1cm) proximal hyperplastic polyp or cancer. An advanced adenoma was defined as an adenomatous polyp > 10 mm in size or with high grade dysplasia or villous elements. Polyp and adenoma prevalence rates were only examined in those patients at average risk for colorectal cancer.

## Results

Of the 34,533 patients who underwent a first colonoscopy during the study period, the quality of the bowel preparation was missing for 118 (6 diabetics). Characteristics of the 34,415 patients included in the analysis are shown in Table 1.

**Table 1:** Study population characteristics.

	Diabetic			Non-Diabetic		
Procedure Year	2013	2014	All	2013	2014	All
n	624 (4%)	1,181 (6%)	1,805 (5%)	15,003 (96%)	17,607 (94%)	32,610 (95%)
**Gender**						
Male	379 (61%)	771 (65%)	1,150 (64%)	6,942 (46%)	8,807 (50%)	15,749 (48%)
Female	245 (39%)	410 (35%)	655 (36%)	8,061 (54%)	8,800 (50%)	16,861 (52%)
**Age Group**						
18–39	0 (0%)	0 (0%)	0 (0%)	134 (1%)	191 (1%)	325 (1%)
40–49	16 (3%)	22 (2%)	38 (2%)	884 (6%)	1,059 (6%)	1,943 (6%)
50–64	437 (70%)	739 (63%)	1,176 (65%)	11,640 (78%)	13,049 (74%)	24,689 (76%)
65+	171 (27%)	420 (36%)	591 (33%)	2,345 (16%)	3,308 (19%)	5,653 (17%)
**Indication**						
Average Risk	396 (63%)	524 (44%)	920 (51%)	9,484 (63%)	8,417 (48%)	17,901 (55%)
FIT+	0 (0%)	333 (28%)	333 (18%)	0 (0%)	3,152 (18%)	3,152 (10%)
Family History	128 (21%)	161 (14%)	289 (16%)	3,673 (24%)	3,930 (22%)	7,603 (23%)
Personal History	75 (12%)	152 (13%)	227 (13%)	1,422 (9%)	1,932 (11%)	3,354 (10%)
Other	25 (4%)	11 (6%)	36 (2%)	424 (3%)	176 (1%)	600 (2%)
**Appointment Time**						
7:30–9:00 AM	495 (79%)	62 (5%)	557 (31%)	3,464 (23%)	4,850 (28%)	8,314 (26%)
9:30 AM or later	129 (21%)	1,119 (95%)	1,248 (69%)	11,539 (77%)	12,757 (72%)	24,296 (75%)
**Findings**						
Any polyp	365 (58%)	779 (66%)	1,144 (63%)	7,519 (50%)	9,631 (55%)	17,150 (53%)
Any screen-relevant lesion	269 (43%)	603 (51%)	872 (48%)	5,189 (35%)	7,204 (41%)	12,393 (38%)
Any advanced adenoma	57 (9%)	152 (13%)	209 (12%)	940 (6%)	1,464 (8%)	2,404 (7%)

In 2013, 79% of diabetics were scheduled for their procedure before 9:30 AM. In 2014, 95% of diabetics were scheduled for their procedure at 9:30 AM or later. Overall, 98% of all patients received a polyethylene glycol-based preparation. The quality of the bowel preparation for procedures performed in 2013 and 2014 are shown in Table 2. From 2013 to 2014, the proportion of diabetics with an inadequate preparation decreased from 7.7% to 3.2% (95% CI for the difference 2.2%–6.8%, P < 0.00005). In contrast there was no significant change in the proportion of non-diabetics with an inadequate preparation (1.9% versus 1.7%; 95% CI for the difference 0.0%–0.6%, P = 0.07). In 2014, the proportion of non-diabetics with an inadequate bowel preparation who were scheduled for a procedure before 9:30 AM was 2.5%, whereas in those scheduled 9:30 AM or later the proportion with an inadequate preparation was 1.4% (95% CI for the difference 0.7%–1.6%, P<0.0005). In logistic regression models that adjusted for the independent effects of age and gender, the odds ratio for an inadequate preparation for diabetics fell from 4.0 (95% CI 2.9–5.5) in 2013 to 1.7 (95% CI 1.2–2.5) in 2014 (Table 3).

**Table 2:** Bowel preparation quality.

	Diabetics			Non-Diabetics		
	2013	2014	Difference 95% Confidence Interval	2013	2014	Difference 95% Confidence Interval
**Adequate-Clean**	286 (45.8%)	798 (67.6%)	—	10,751 (71.7%)	13,019 (73.9%)	—
**Adequate-Stool**	290 (46.5%)	345 (29.2%)	—	3,961 (26.4%)	4,294 (24.4%)	—
**Inadequate**	48 (7.7%)	38 (3.2%)	2.2–6.8% (P<0.00005)	291 (1.9%)	294 (1.7%)	0.0–0.6% (P = 0.07)

**Table 3:** Multivariate predictors of inadequate bowel preparation.

	Odds Ratio	95% Confidence Interval
**2013 Procedures**		
**Gender**		
Male	reference	—
Female	0.98	0.79–1.22
**Age Group**		
18–39	reference	—
40–49	1.07	0.24–4.72
50–64	1.22	0.30–4.98
65+	1.82	0.44–7.51
**Diabetic**		
No	reference	—
Yes	3.98	2.89–5.48
**2014 Procedures**		
**Gender**		
Male	reference	—
Female	0.96	0.77–1.19
**Age Group**		
18–39	reference	—
40–49	0.87	0.19–4.00
50–64	1.48	0.36–5.99
65+	2.37	0.58–9.70
**Diabetic**		
No	reference	—
Yes	1.75	1.24–2.48

Other procedure outcomes for diabetics in 2014 and 2015 are shown in Table 4.

**Table 4:** Rates of other procedure outcomes in diabetic patients.

	2013	2014	95% Confidence Interval for the Difference
**All Diabetics**^**1**^			
**Incomplete**	13 (2.1%)	17 (1.4%)	-0.7–2.0% (P = 0.3)
**Mean Duration (minutes)**			
**All procedures**	18	19	-1.4–0.4 (P = 0.3)
**Procedures with no polyps**	15	15	-0.7–1.4 (P = 0.5)
**Average Risk Diabetics Only (****n** **= 920)**			
**Any polyp**	220 (56%)	294 (56%)	-5.9–7.0% (P = 0.9)
**Any screen-relevant lesion**	163 (41%)	215 (41%)	-6.6–6.3% (P = 0.9)
**Any advanced adenoma**	28 (7%)	47 (9%)	-1.6–5.4% (P = 0.3)

## Discussion

It is widely accepted that splitting the dose of a bowel preparation with the second dose given within six hours of the procedure is superior to administering all of the preparation the day before the procedure.[9, 13] For patients undergoing an early morning procedure, this requires the patient to wake during the night to finish the preparation. However, for many patients the inconvenience of completing a preparation during the middle of the night could dissuade them from accepting an early morning appointment, even if they understood the importance of a good preparation. In one survey of patients who were scheduled for an early morning colonoscopy with a split-dose preparation, 22% did not get up during the night to take the second dose of the preparation[14] In our clinic setting, where procedures are largely elective in nature, our anecdotal experience is that patients delay their colonoscopy to a later date rather than accept an earlier appointment that requires them to wake during the night to take the preparation.

Our Centre changed how diabetic patients were scheduled for colonoscopy after a quality improvement audit identified that diabetic patients had a higher risk for inadequate bowel preparation. Prior to the audit diabetic patients were preferentially scheduled for colonoscopy in an early morning spot (7:30–9:00 AM). This resulted in the majority of diabetic patients taking all of the preparation the day before the procedure. After the audit, diabetic patients were scheduled for appointments no sooner than 9:30 AM to allow them to complete a split-dose preparation without waking before 4:30 AM.

Our results show that with this change, the risk of a diabetic patient having an inadequate preparation decreased from 8% to 3%. This difference persisted when we controlled for patient gender and age. When qualitatively examining the distribution of bowel preparation scores for diabetics, the distribution was more in keeping with that observed for non-diabetic patients. Importantly, the preferential scheduling of diabetics did not have the unintended consequence of clinically meaningful higher rates of inadequate preparation among non-diabetic patients. However, the improvement in overall preparation quality among diabetics did not translate into significant improvements in other procedure outcomes, such as rates of procedures complete to the cecum, overall duration of the procedure or detection rates of polyps or adenomas.

Endoscopy units are challenged by how to best manage patients undergoing early morning procedures. Options include (1) scheduling gastroscopies or other procedures not requiring bowel preparation in those spots, (2) not offering patients a choice in appointment scheduling, (3) mandating that patients complete the preparation during the night and (4) not splitting the preparation. Option 1 is not feasible in units that predominantly perform colonoscopies. Option 2 goes against the principles of patient-centered care in endoscopy as outlined in the Global Rating Scale for Endoscopy.[15] Option 3 may risk non-compliance and may not be safe or feasible in patients who are frail or require assistance with taking the bowel preparation or accessing a toilet.[14]

Another option, and the one we elected to employ, is to identify patients at higher risk of a poor bowel preparation and preferentially scheduling them at a time that allows a split dose preparation to be used safely and conveniently. Despite clear evidence that a split dose preparation is superior, many patients who take all of the preparation the day before have adequate bowel cleanliness at colonoscopy. For example, a crude analysis of the 10 analyzable trials included in the meta-analysis of Martel et al. of split-dose versus day-before PEG regimens shows that approximately 50% of patients who received day-before PEG had adequate preparations.[13] Therefore, a potential solution to requiring all patients to complete a split-dose preparation, regardless of appointment time, is to identify those patient less likely to achieve an adequate preparation with a day-before preparation and ensure that they receive an appointment time that facilitates completion of a split-dose preparation. Using an algorithm to identify at risk patients and provide individualized, risk-based bowel preparations is not novel,[16] but there is limited to no data to support this approach. We believe that our findings validate this approach and should drive researchers to further study and refine individualized approaches to preparation for colonoscopy.

The major limitation of this study is the use of an unvalidated bowel preparation scale. Although the scale was recommended by an expert panel, it remains unvalidated. However, in our setting it is directly tied to patient management, as the expectation would be that a patient with an inadequate preparation would require a repeat colonoscopy or some other screening test. Therefore, we believe our scale does provide valid data in regards to whether the procedure was considered an adequate screening procedure. In addition, our centre performs only outpatient procedures and only sees patients without significant medical comorbidities. This means that the diabetics in our group did not suffer from advanced end-organ complications, such as gastroparesis or severe gastrointestinal dysmotility or renal dysfunction. Therefore, our results should be seen as applying only to generally well diabetics in an ambulatory setting. Finally, we do not have comprehensive data on diabetes-related events, such as hypoglycemia. But we are unaware of any significant adverse events related to our scheduling changes.

In conclusion, we have found that preferentially scheduling diabetic patients later in the morning that allowed for a split dose bowel preparation to be safely and conveniently completed resulted in decreased rates of inadequate bowel preparations without disadvantaging other patients. Other colonoscopy outcomes (procedure duration, lesion detection) were not improved. Endoscopy units should consider risk stratifying their patients in regards to risk factors for poor preparation and targeting specific interventions at those at increased risk.

## References

[1] Chung YW, Han DS, Park KH, Kim KO, Park CH, Hahn T, et al Patient factors predictive of inadequate bowel preparation using polyethylene glycol: a prospective study in Korea. Journal of Clinical Gastroenterology. 2009;43(5):448–52. doi: 10.1097/MCG.0b013e3181662442 . 18978506 10.1097/MCG.0b013e3181662442

[2] Taylor C, Schubert ML. Decreased efficacy of polyethylene glycol lavage solution (golytely) in the preparation of diabetic patients for outpatient colonoscopy: a prospective and blinded study. American Journal of Gastroenterology. 2001;96(3):710–4. doi: 10.1111/j.1572-0241.2001.03610.x . 11280539 10.1111/j.1572-0241.2001.03610.x

[3] Hassan C, Fuccio L, Bruno M, Pagano N, Spada C, Carrara S, et al A predictive model identifies patients most likely to have inadequate bowel preparation for colonoscopy. Clinical Gastroenterology & Hepatology. 2012;10(5):501–6. doi: 10.1016/j.cgh.2011.12.037 . 22239959 10.1016/j.cgh.2011.12.037

[4] Nguyen DL, Wieland M. Risk factors predictive of poor quality preparation during average risk colonoscopy screening: the importance of health literacy. J. 2010;19(4):369–72. . 21188326

[5] Dik VK, Moons LM, Huyuk M, van der Schaar P, de Vos Tot Nederveen Cappel WH, Ter Borg PC, et al Predicting inadequate bowel preparation for colonoscopy in participants receiving split-dose bowel preparation: development and validation of a prediction score. Gastrointestinal Endoscopy. 2015;81(3):665–72. doi: 10.1016/j.gie.2014.09.066 . 25600879 10.1016/j.gie.2014.09.066

[6] Froehlich F, Wietlisbach V, Gonvers JJ, Burnand B, Vader JP. Impact of colonic cleansing on quality and diagnostic yield of colonoscopy: the European Panel of Appropriateness of Gastrointestinal Endoscopy European multicenter study. Gastrointestinal Endoscopy. 2005;61(3):378–84. 15758907 10.1016/s0016-5107(04)02776-2

[7] Harewood GC, Sharma VK, de Garmo P. Impact of colonoscopy preparation quality on detection of suspected colonic neoplasia. Gastrointestinal Endoscopy. 2003;58(1):76–9. doi: 10.1067/mge.2003.294 . 12838225 10.1067/mge.2003.294

[8] Kim ER, Sinn DH, Kim JY, Chang DK, Rhee PL, Kim JJ, et al Factors associated with adherence to the recommended postpolypectomy surveillance interval. Surgical Endoscopy. 2012;26(6):1690–5. doi: 10.1007/s00464-011-2094-2 . 22437946 10.1007/s00464-011-2094-2

[9] Johnson DA, Barkun AN, Cohen LB, Dominitz JA, Kaltenbach T, Martel M, et al Optimizing adequacy of bowel cleansing for colonoscopy: recommendations from the US Multi-Society Task Force on Colorectal Cancer. American Journal of Gastroenterology. 2014;109(10):1528–45. doi: 10.1038/ajg.2014.272 . 25223578 10.1038/ajg.2014.272

[10] Perioperative management of blood glucose in adults with diabetes mellitus [Internet]. 2016 [cited 01/14/2017]. Available from: www.uptodate.com/contents/perioperative-management-of-blood-glucose-in-adults-.

[11] Lieberman D, Nadel M, Smith RA, Atkin W, Duggirala SB, Fletcher R, et al Standardized colonoscopy reporting and data system: report of the Quality Assurance Task Group of the National Colorectal Cancer Roundtable. [Review] [32 refs]. Gastrointestinal Endoscopy. 2007;65(6):757–66. doi: 10.1016/j.gie.2006.12.055 17466195 10.1016/j.gie.2006.12.055

[12] Lieberman DA, Rex DK, Winawer SJ, Giardiello FM, Johnson DA, Levin TR, et al Guidelines for colonoscopy surveillance after screening and polypectomy: a consensus update by the US Multi-Society Task Force on Colorectal Cancer. [Review]. Gastroenterology. 2012;143(3):844–57. doi: 10.1053/j.gastro.2012.06.001 22763141 10.1053/j.gastro.2012.06.001

[13] Martel M, Barkun AN, Menard C, Restellini S, Kherad O, Vanasse A. Split-Dose Preparations Are Superior to Day-Before Bowel Cleansing Regimens: A Meta-analysis. Gastroenterology. 2015;149(1):79–88. doi: 10.1053/j.gastro.2015.04.004 . 25863216 10.1053/j.gastro.2015.04.004

[14] Unger RZ, Amstutz SP, Seo DH, Huffman M, Rex DK. Willingness to undergo split-dose bowel preparation for colonoscopy and compliance with split-dose instructions. Digestive Diseases & Sciences. 2010;55(7):2030–4. doi: 10.1007/s10620-009-1092-x . 20082216 10.1007/s10620-009-1092-x

[15] Sint NJ, de JV, de Man RA, ter BF, Cahen DL, Moolenaar W, et al The Global Rating Scale in clinical practice: a comprehensive quality assurance programme for endoscopy departments. Digestive & Liver Disease. 2012;44(11):919–24. 22840567 10.1016/j.dld.2012.06.021

[16] Rex DK. Bowel preparation for colonoscopy: entering an era of increased expectations for efficacy. Clinical Gastroenterology & Hepatology. 2014;12(3):458–62. doi: 10.1016/j.cgh.2013.11.003 . 24239858 10.1016/j.cgh.2013.11.003

